# Swimming behavior of emigrating Chinook Salmon smolts

**DOI:** 10.1371/journal.pone.0263972

**Published:** 2022-03-15

**Authors:** Rusty C. Holleman, Edward S. Gross, Michael J. Thomas, Andrew L. Rypel, Nann A. Fangue

**Affiliations:** 1 Center for Watershed Sciences, University of California Davis, Davis, California, United States of America; 2 Department of Civil and Environmental Engineering, University of California Davis, Davis, California, United States of America; 3 Department of Wildlife, Fish and Conservation Biology, University of California Davis, Davis, California, United States of America; Institut de Recherche pour le Developpement, FRANCE

## Abstract

Swimming behavior of Chinook Salmon (*Oncorhynchus tshawytscha*) smolts affects transit time, route selection and survival in complex aquatic ecosystems. Behavior quantified at the river reach and junction scale is of particular importance for route selection and predator avoidance, though few studies have developed field-based approaches for quantifying swimming behavior of juvenile migratory fishes at this fine spatial scale. Two-dimensional acoustic fish telemetry at a river junction was combined with a three-dimensional hydrodynamic model to estimate in situ emigration swimming behavior of federally-threatened juvenile Chinook salmon smolts. Fish velocity over ground was estimated from telemetry, while the hydrodynamic model supplied simultaneous, colocated water velocities, with swimming velocity defined by the vector difference of the two velocities. Resulting swimming speeds were centered around 2 body lengths/second, and included distinct behaviors of positive rheotaxis, negative rheotaxis, lateral swimming, and passive transport. Lateral movement increased during the day, and positive rheotaxis increased in response to local hydrodynamic velocities. Swim velocity estimates were sensitive to the combination of vertical shear in water velocities and vertical distribution of fish.

## Introduction

Chinook Salmon (*Oncorhynchus tshawytscha*) populations across the Pacific Coast declined throughout the 20th century, and have remained low in the Central Valley of California in particular [1, 2]. Of the four recognized evolutionary significant units (ESUs) of Chinook Salmon in the Central Valley, two (winter-run and spring-run, named for the season when adults return to freshwater; [3]) are listed under the federal Endangered Species Act. Chinook Salmon exhibit anadromous life-histories, hatching and rearing in fresh water before emigrating to the ocean as juveniles. In the Central Valley, juveniles experience high mortality during emigration, due largely to geomorphic and hydrologic changes at the watershed scale that have degraded or eliminated habitat, as well as decreased river flows due to drought and water exports, entrainment in water diversions, predation by non-native species, decreased food supply, and presence of contaminants [3–5]. Survival varies strongly as a function of water year hydrology with high-flow, wet years producing higher survivorship and low-flow, drought years lower survivorship [6, 7]. Emigration survival rates in California’s Central Valley are notably lower compared to parallel populations at more northern latitudes; thus identifying management actions aimed at improving survivorship of juvenile salmon is central to salmon conservation.

In an ongoing experimental reintroduction of the San Joaquin spring-run ecologically significant unit (ESU), hatchery-reared juveniles are released approximately halfway down the original migratory corridor. Emigrating smolts must transit the remaining migratory corridor of the mainstem river and the Delta’s bifurcations and branches, prior to reaching San Francisco Bay. At the first such bifurcation, henceforth “Head of Old River,” smolts are routed either to the west (along Old River) or east (along the San Joaquin River). Understanding drivers of behavior and consequent routing probabilities at critical junctions such as the Head of Old River is of interest to both fish and water managers as route selection can lead to very different survival outcomes [5, 8].

Studies of juvenile salmonid swimming can be broadly grouped into studies of swimming performance and swimming behavior. Swimming performance refers to the physiological capacity for swimming, generally the maximum speed for a prescribed duration. Swimming behavior can be defined as how an individual selects among physiologically possible swimming actions, potentially influenced by life stage and environmental cues [9, 10].

Swimming performance has been a research focus in salmonids since the pioneering work of [11] and remains an important tool for understanding stressors [12], habitat adaptation [13, 14], tag burden [15, 16] and effects of disease [17]. These studies often measure maximum burst, sustained swimming speeds, or critical swimming speeds (*U*_*crit*_) of fish in flumes and *in situ* swim tunnels [18–20]. Specific to Chinook Salmon [21], studied instantaneous swimming speed of Chinook Salmon smolts on the San Joaquin River several kilometers downstream of our study area. They observed that the maximum swimming speed decreased with increased turbidity and temperature, and was typically in the range of 5–7 body lengths per second (BL s^-1^) for fish 71–99 mm fork length (approximately 0.51 m s^-1^). Swim tunnel studies are mostly limited to positive rheotaxis (swimming against the flow), with the exception of swimming in annular flumes where negative rheotaxis (swimming with the flow) can also be observed (e.g., [9]).

Swimming behaviors include foraging, holding, positive rheotaxis, negative rheotaxis, and passive behavior (i.e. an absence of swimming resulting in movement exactly with the speed of the surrounding water). Studies of swimming behavior can be further categorized in terms of the spatial and temporal scales of the study, and the life stages of the individuals in the experiment. At the landscape or basin scale, behavior generally cannot be observed directly, but is inferred from large scale movement of animals, such as in [22] where timing of emigration was linked to basin hydrology. Behavior at the scale of river junctions, and the associated route selection, is of great practical interest and has been successfully related to local hydrodynamic forcing in studies such as [23, 24]. In particular [24], correlated lateral position of fish (relative to the critical streakline) to route selection 250 m downstream, which can be seen as a test of the persistence of cross-sectional distributions. Correlation was strong during high flows, but weakened for low flows, which was attributed to lateral swimming. At the reach scale [25], linked holding versus foraging behaviors of pre-emigration juvenile Chinook Salmon to time of day, generally finding foraging at night and holding during the day. At yet smaller spatial scales, behavioral responses have been linked to features such as turbulent flow [26], predators [27, 28] and prey [26].

At the reach scale, methods that combine acoustic tracking and hydrodynamic modeling can be used to study swimming behaviors, fish reactions to environmental stimuli, and effects of swimming behavior on in-river movement and route selection. These methods have been applied to passage of fish through dams [29, 30], fish screens [31] and route selection in channelized systems [32]. Particle- and agent-based modeling studies such as [30, 32] test the implications of hypothesized swimming behaviors [30]. Hypothesized a set of four behaviors—downstream-biased random walk, swim to greater water velocity, swim upstream, and vertical swimming to minimize pressure changes—along with utility functions based on pressure and acceleration that guided the moment-to-moment activation of specific behaviors. These behaviors were encoded in an agent-based model and calibrated to reproduce observed route selection [32]. Established links between one hypothesized behavior (bias toward the surface) and route selection, complementing studies such as [7, 33] linking route selection and survival in salmonids [31]. Took a correlative approach to swimming behavior, pairing acoustic tracking data and a hydrodynamic model to obtain swimming velocities at short time scales in the vicinity of a turbine intake. They found correlations between swimming velocity (as speed and directional persistence) and hydrodynamic variables including water velocity and turbulent kinetic energy.

Of these previous studies at similar spatial scales, the present study has many goals in common with [31], and similarly pairs a three-dimensional hydrodynamic model with fish tracks derived from acoustic telemetry with the goal of quantifying swimming behavior at short time scales. The present study expands on quantifying swimming behavior by (i) extending hydrodynamic model calibration to the full three-dimensional flow, (ii) considering uncertainty in swim velocity associated with hydrodynamic error and uncertainty in vertical position of fish, (iii) examining turbidity and time of day as covariates of swimming behavior, and (iv) considering a different species (Central Valley spring-run Chinook Salmon rather than Atlantic Salmon *Salmo salar*).

The goals of this study were to observe the swimming behavior of emigrating Chinook Salmon smolts and how this behavior is related to environmental variables. This information is critical to understanding (i) how modifications to junction and reach geometries affect fish passage, (ii) how persistent or transient cross-sectional distributions are, and (iii) how to realistically parameterize fish swimming in particle-tracking models. These goals were achieved by combining high-resolution telemetry data from a dense array of hydrophones with water velocity predictions from a hydrodynamic model to obtain instantaneous, *in situ* estimates of smolt swimming. The hydrodynamic model was calibrated to a level enabling not just correlative analysis of behaviors but also quantitative descriptions of swimming that could be directly utilized in individual-based models. These results provided new insights into the overall transport process of smolts during emigration, with implications for management strategies to improve survival in systems such as Sacramento-San Joaquin Delta.

## Materials and methods

### Site description

**Fig 1:**
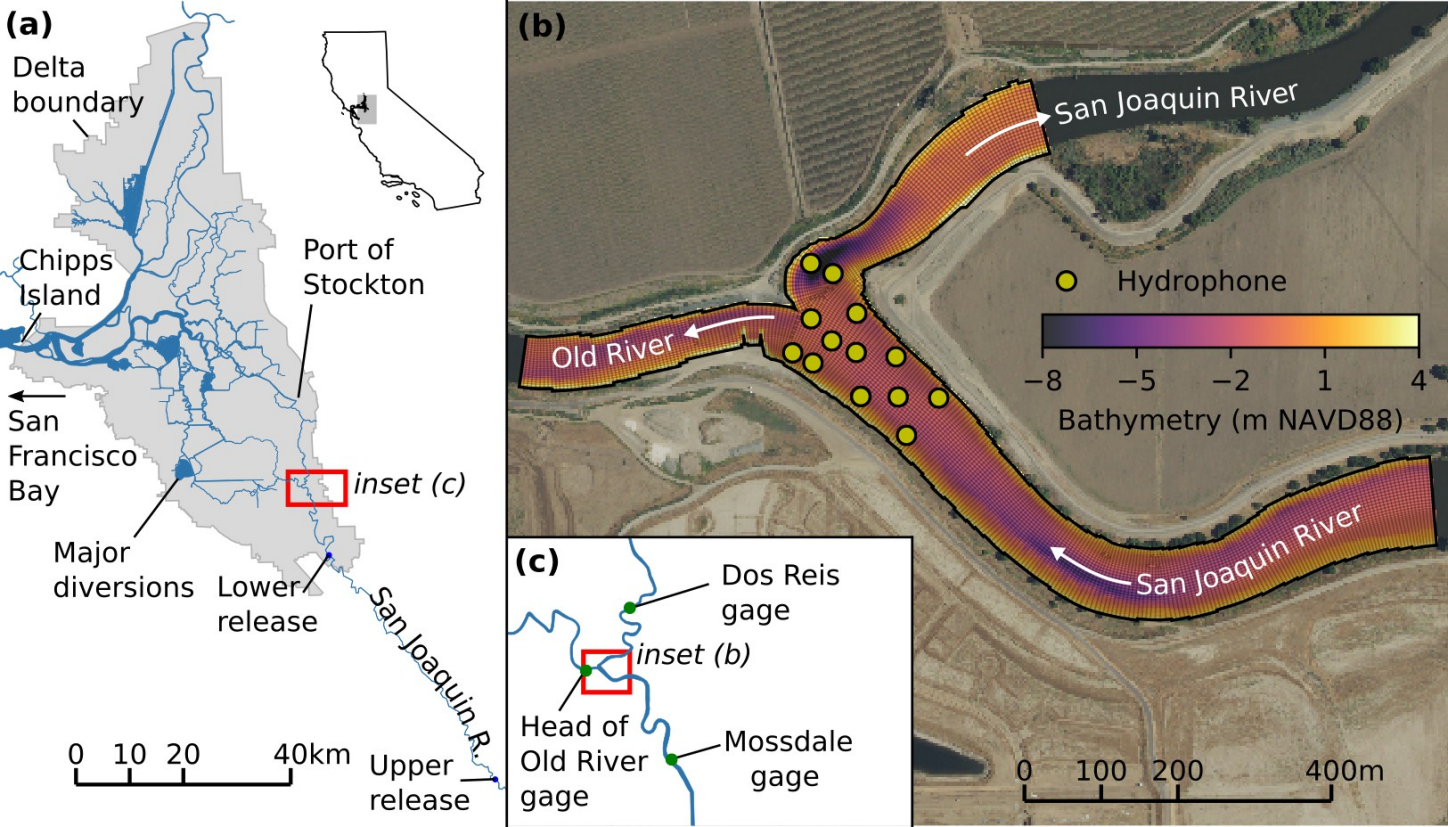
Study site. (a) Sacramento-San Joaquin Delta, including San Joaquin River and fish release locations. (b) Head of Old River study site, with computational grid and bathymetry, and the layout of the hydrophone array. White arrows show the downstream flow direction (noting that tidal flow reversal is possible on the downstream section of the San Joaquin River). (c) Location of gages relative to study site.

Our study area was the junction of the San Joaquin River and the head of Old River. This diffluence represents the southeastern entry point to the Sacramento-San Joaquin Delta and the landward end of the northern San Francisco Estuary (Fig 1). Flows at this junction have substantial tidal variation when river discharge is small, with decreasing tidal influence as discharge increases. Net flows down Old River are typically slightly greater than net flows into the San Joaquin River side of the junction. During typical flow conditions, most water entering Old River from the San Joaquin River is removed at the major diversions—the C.W. Bill Jones Pumping Plant of the Central Valley Project (CVP), operated by the U.S. Bureau of Reclamation, and the Harvey O. Banks Pumping Plant of the State Water Project (SWP), operated by the California Department of Water Resources. Correspondingly, fish routed through Old River are at high risk of entrainment at the water diversion facilities. To mitigate these risks, fish salvage facilities, colocated with the pumping plants, collect fish ahead of the pumps. These fish are transported over land to Chipps Island at the western boundary of the Delta, and released back into the Estuary [5]. Fish emigrating along the San Joaquin River route, rather than Old River, traverse the engineered channels of the Delta, including the deepwater Port of Stockton, before potentially exiting the Delta at Chipps Island.

The study area is shallow, with average water depths of 3–4 m, with the exception of a deep scour hole on the San Joaquin River immediately downstream of the junction. The substrate is primarily sand, with sand waves in portions of the channel. Near the shoreline and particularly on the outsides of bends, the substrate includes riprap cobbles. In low-flow and moderate-flow years a temporary rock barrier is installed from March to May across Old River, immediately downstream of the diffluence. When installed the barrier is 25 m wide, extends across the channel, and routes smolts and river flow into the San Joaquin River instead of Old River. This minimizes smolt entrainment in agricultural diversions in the South Delta (including the aforementioned pumping plants), and serves to improve water quality in the downstream San Joaquin River [34]. In 2018 the structure was partially installed, extending from the southern shoreline and blocking a portion of the channel.

**Fig 2:**
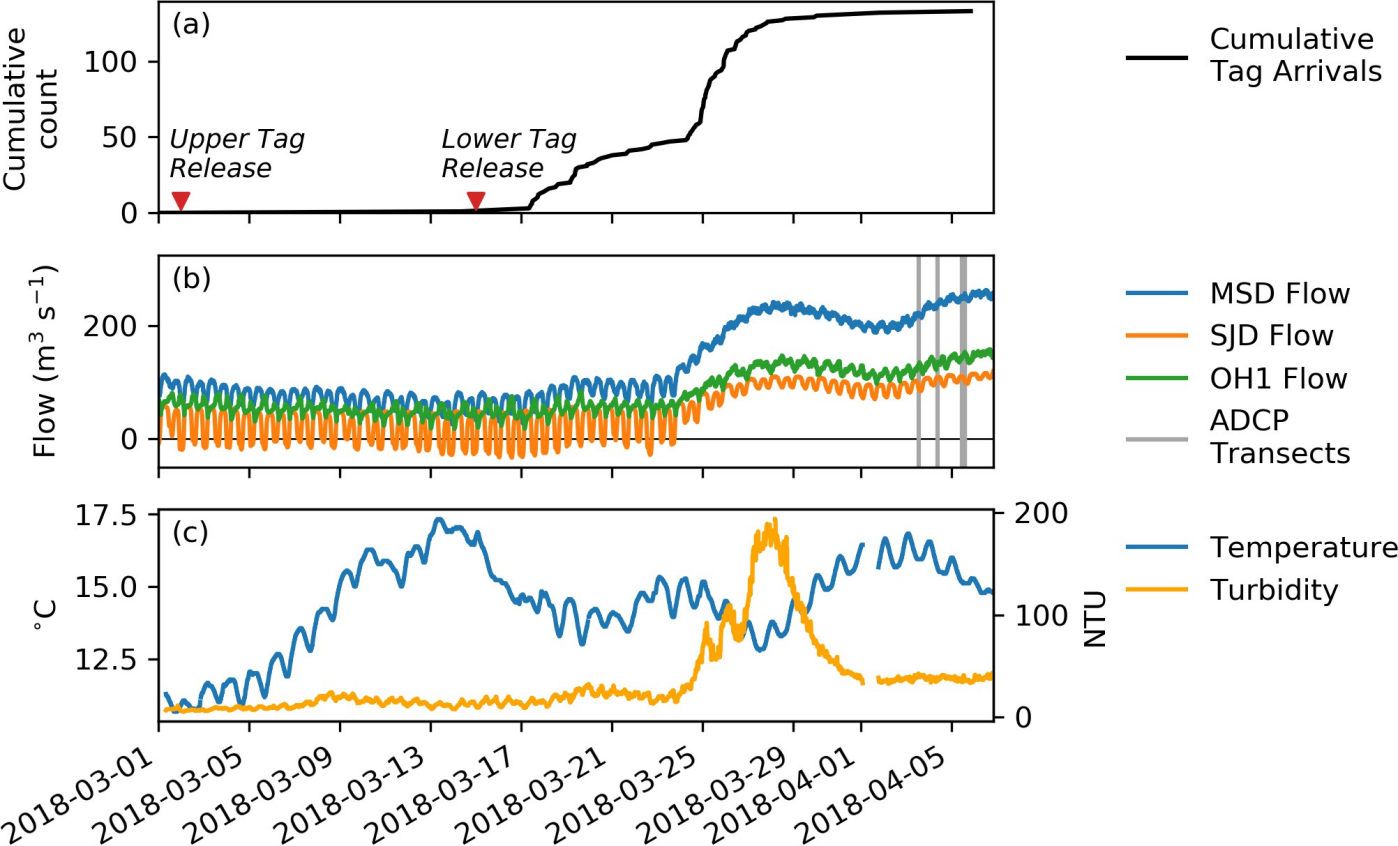
Timeline. Chronology (year-month-day) of (a) fish releases, cumulative tag detections, (b) hydrograph and ADCP data collection, and (c) temperature and turbidity. Flows are shown for Mossdale (MSD), above the junction, San Joaquin Dos Reis (SJD), below the junction on the San Joaquin River, and Old River at Head (OH1), below the junction on Old River.

Continuous water flow data is available on the San Joaquin River at the Mossdale gage (California Water Data Library (WDL) station B95820Q) upstream of the junction, downstream of the junction at Dos Reis (WDL B95760) and at the Head of Old River (WDL B95400Q), as shown on Fig 2. During the March, 2018–April, 2018 study period a tidal signal was clear at all stations, though the flow reversed only below the junction on the San Joaquin River at Dos Reis, and there only before flows began to increase around March 24.

### Fish tagging, release, and telemetry

A total of 650 hatchery-reared smolts were released from two sites upstream of the HOR study site: an upstream site, 60 km upstream of the study site between Stevinson, CA and Gustine, CA; and a downstream site, 14 km upstream of the study site near Durham Ferry, CA (Fig 1A). While fish from the upstream release traversed more of the historic migratory route down the San Joaquin River, the potential for low in-river survival along this stretch (47% for a similar reach in 2009, [5]) led to the addition of the lower release site in order to ensure that sufficient numbers of fish reached the study site. Releases occurred in March, 2018, timed to coincide with the historical emigration season for sub-yearling, spring-run juveniles [3], with the upper release of 325 smolts occurring on March 2, 2018 and the lower release of 325 individuals on March 15, 2018. Real-time data from acoustic nodes between the release sites allowed us to time the lower release to coincide with individuals arriving from the upper release.

Salmon smolts used in this study originated from the experimental population of spring-run Chinook reared at the Salmon Conservation and Research Facility (SCARF), operated by the California Department of Fish and Wildlife and located at the base of Friant Dam, near Fresno, California. Smolts were reared at SCARF until fish reached sizes sufficient to maintain a tag burden of ≤ 5% of total body weight, a minimum of 4.2 g for a 0.216 g tag [35]. Smolt lengths and weights were measured at the time of acoustic tagging. Smolt length averaged 76.6 mm fork length (FL), with a range of 71–86 mm FL. Although average length by release group differed by less than 1 mm (upper = 76.9 mm, lower = 76.3 mm), the difference was statistically significant (Mann-Whitney U test, W = 71484, p = 0.0001) with individuals from the upper release being larger.

Acoustic tagging of the smolts was performed by intraperitoneal implantation where a 2–3 mm incision was made 0.5–1 mm off the ventral midline, anterior to the pelvic girdle. After each tag was inserted the incision was closed with a single 2x2 surgeon’s knot (6/0 PDS II suture). Prior to surgeries, fish were anesthetized with a buffered, 90-mg/l solution of tricaine methanesulfonate (MS-222; Argent Chemical, Redmond, Washington). Additional surgery details and information on handling and anesthesia were similar to those described in [36]. Tagging was performed under ESA Section 10(a)(1)(A) permit #20571, and adhered to the policies of University IACUC #21614. Tagged fish were held and observed at SCARF for 3–5 days before release for the upper release, and 10–12 days before release for the lower release, during which time no mortalities were observed. Given the small sizes of juvenile salmon, we utilized the smallest Juvenile Salmon Acoustic Tracking System (JSATS) acoustic transmitter commercially available (model SS400, ATS Issanti, MN). Transmitters weighed 216 mg and measured 15 mm in length and 3.38 mm in diameter. Tags were configured with a 5 second pulse rate interval (PRI) for an estimated tag battery life of 90 days. We transported all tagged salmon in aerated live wells from the tagging location to the release sites, where fish underwent temperature acclimation prior to release. The acclimation procedure involved water exchange from the transport tank with river water at a rate that limited temperature change to 2°C per hour until ambient river water temperatures were obtained.

A 416 kHz multi-dimensional positioning system (Teknologics, LLC., Edmonds, WA), composed of an array of 13 hydrophones nominally spaced 70 m apart, was used to record pings from the tags. In order to constrain clock drifts, where possible we used cellular-enabled cabled units, configured to resynchronize the internal clock every 24 h (n = 6). The remaining hydrophones (n = 7) were configured to be autonomous. Divers positioned each hydrophone unit on a 1–2m, #5 rebar rod driven into the river bottom. Each mooring rod was also weighted with 34 kg of steel and a back-up tether to the hydrophone. Once the hydrophones were in place, a survey rod was lowered to the diver and three GPS coordinates were measured at the tip of the hydrophone with a Trimble R10 network-corrected GPS providing <2 cm of position error in the horizontal (Trimble Inc. Sunnyvale, CA). Internal angles for triples of adjacent hydrophones had a median of 53° and inter-quartile range of 43–75°. Distance between adjacent hydrophones had an inter-quartile range of 49–65 m with a median of 53 m.

Multilateration (the estimation of tag positions from detected pings) utilized the YAPS software [37]. Position estimates did not include a vertical coordinate as the shallow water column did not provide enough vertical separation between hydrophones to discern vertical position. Compared to methods that process pings individually, YAPS estimates all positions and error terms along a track simultaneously using a maximum likelihood approach. The likelihood function includes terms for multipath error (e.g. if the acoustic signal reflects off the water surface), autocorrelation via a random walk model, and an estimate of jitter in ping intervals. In some cases YAPS can recover usable position estimates even when only two hydrophones recorded a particular ping. When a tag was detected by a single hydrophone multiple times over a short period (relative to the ping interval), the latter detections were assumed to arise from signal reflections from the riverbed or water surface. These latter detections were discarded before processing with YAPS.

A critical step in multilateration is synchronization of the hydrophone clocks, which can be challenging in noisy environments where hydrophones do not reliably detect synchronization pings from other hydrophones. YAPS estimates a time-varying clock offset (as a piecewise quadratic function) for each hydrophone while simultaneously estimating receiver positions. When hydrophones reliably detect each other, this approach is robust and precise. In the present study the detection efficiency for synchronization pings was often low, likely due to noise and acoustic path constraints in shallow and energetic flow. In some cases this led to discontinuities in clock offset functions, and exposed non-determinism in the fitting procedure whereby repeated analyses of the same inputs yielded different outputs, complicating the analysis work flow. To avoid these issues we implemented an additional clock synchronization step beyond the typical YAPS process (source code available at https://github.com/rustychris/sync_pings_linear). We used the YAPS synchronization approach to estimate hydrophone locations, and then took those positions as fixed and solved for the clock offsets outside of YAPS. This external solution approach is greatly simplified by assuming fixed station locations (the problem can be reduced to a least-squares solution to a system of linear equations). Similar to the standard approach, clock offsets are defined as piecewise functions, but using a continuous, piecewise linear function for each hydrophone as opposed to a discontinuous piecewise quadratic function (higher order interpolants were tested but did not improve error metrics). Residuals (expected ping timestamps—measured ping timestamps) were used for detection of erroneous pings, iteratively solving the system and, as long as the root-mean-square (RMS) residual was above 1 ms, removing the ping with the worst residual and solving again. Pings were processed in five-day periods to balance continuity and computational efficiency. A typical five-day period had 500,000 pings, 30 erroneous pings, took approximately 30 s to calculate clock offsets, and had a final RMS residual of 0.4 ms.

Positions and tracks were filtered based on YAPS-reported error metrics, track length, overlap with the study area, and transit time through the study area. In addition to position, YAPS calculates an estimate of position uncertainty for each ping. Tag positions for which the estimated standard deviation exceeded 10 m or the number of receivers was fewer than two were eliminated. Each tag’s track was truncated to start and end with solutions utilizing at least three hydrophones. Tags with fewer than 10 detected positions were omitted from further analysis. Based on average water velocities through the study area, we calculated that in the absence of swimming a tag would transit the array in 15–30 min. Tags that were observed in the array for over 60 min were considered likely predators and omitted from further analysis. Positions below the junction were omitted from further analysis due to lack of robust hydrodynamic data (described below). Positions more than 30 m upstream of the start of the receiver array were also eliminated.

### Hydrodynamics

**Fig 3:**
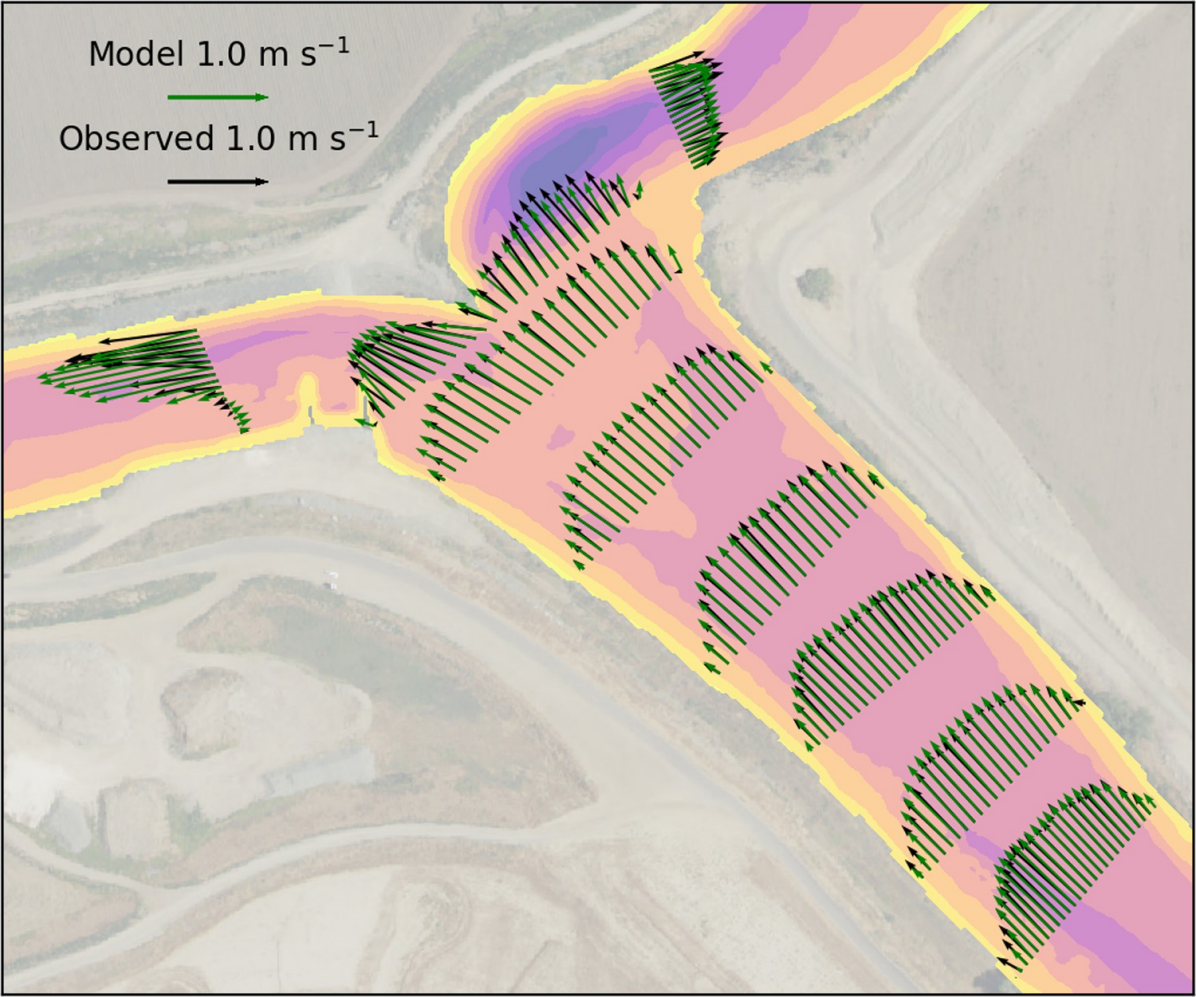
Location of water velocity measurements and model–data comparisons. Velocity vectors in the horizontal for each transect showing modeled (green) and observed (black) velocities averaged over the top 2 m of the water column.

Velocity data were collected by a shipboard acoustic doppler current profiler (ADCP; M9, Sontek, Inc., San Diego, CA) for the purpose of hydrodynamic model calibration. The ADCP was operated in adaptive frequency mode, automatically switching between 3 MHz pings in near-shore areas and 1 MHz pings in deeper water. Blanking distance was 0.20 m, and vertical bin size ranged 0.02–0.50 m. Pings were averaged in the Sontek RiverSurveyor software over 1 s intervals, with 7–35 pings s^-1^. Maximum speed of the survey vessel relative to the riverbed (while sampling) was 0.5 m s^-1^. Ship movement was accounted for with acoustic bottom track. Of the nine transects, six were above the junction, one at the junction, and one transect below the junction on each branch (Fig 3). Additionally, the depth of the ADCP transducer below the water surface was calibrated by matching the ADCP-measured net flow to the Mossdale gauging station data located 4 km upstream. Each transect was repeated six times in succession and the results averaged to reduce noise.

A three-dimensional hydrodynamic model was developed for the study, encompassing a reach of the San Joaquin River and the Head of Old River. The computational grid (Fig 1) has ~11,000 cells in the horizontal with a typical lateral (across-flow) spacing of 2–3 m and longitudinal (along-flow) spacing of 4 m. There are 50 layers in the vertical in a z-layer configuration, evenly spaced at 0.27 m. The model bathymetry was compiled from multibeam bathymetry [38], pre-compiled seamless topobathy for the region [39], ADCP data collected during the field campaign, and an estimate of the footprint of the partially installed temporary barrier based on satellite imagery. The multibeam data was used above the junction, while ADCP-derived bathymetry was used below the junction as it was the only dataset collected while the partial barrier was in place. Each beam of the ADCP depth data was processed independently, providing additional spatial resolution in the deeper areas. Both the ADCP data and the multibeam data were processed with an along-channel anisotropic smoothing method prior to interpolation onto the grid. Multibeam data with a 0.30 m resolution was additionally used to derive a bed roughness parameter. Depth samples were grouped by computational grid cell, detrended, and the remaining root-mean-square variation used as a physical roughness length scale. In the hydrodynamic model this length was scaled by a factor of 1/30 to arrive at a hydrodynamic roughness length (z_0_) [40].

The boundary conditions imposed flows both upstream and downstream on the San Joaquin River, while time-varying water level was prescribed at the Old River boundary. All three boundary conditions utilized observed data obtained from the California Water Data Library. Upstream San Joaquin flows utilized data from the Mossdale gage. Downstream San Joaquin flows utilized flows from the Dos Reis gage, and Old River stage was from the Head of Old River gage.

The SUNTANS numerical model [41] was used for hydrodynamic calculations. Model calibration was evaluated in terms of the velocity bias vector ***b***,

$$b=<m_{i}-o_{i}>$$ (1)

and unbiased root mean square error (uRMSE),

$$uRMSE=\sqrt{<{\left\|m_{i}-o_{i}-b\right\|}^{2}>}$$ (2)

where ***m***_*i*_ denotes modeled velocity vector at the *i*th point along a transect (after averaging over a specific vertical range as discussed below), ***o***_*i*_ the observed velocity vector for the *i*th point along a transect (similarly averaged in the vertical), <*x*> denotes the average of *x* over all *i*, and ‖***y***‖ denotes the magnitude of vector ***y***. Modeled, observed and bias velocities are all vectors in the horizontal plane; vertical velocities were not examined in the observations or model output. Bias is reported separately for the longitudinal and lateral components of the velocity, and uRMSE is reported for the longitudinal component (*u*), lateral component (*v*) and vector magnitude. The calibration process involved adjustments to friction parameters, grid refinement in the horizontal and vertical, and parameters controlling the resolution of vertical advection and diffusion. While the goal of calibration was to reduce both bias and uncorrelated errors quantified by uRMSE, some adjustments decreased one error metric while increasing the other. In such cases we favored decreases in bias over decreases in uRMSE (in terms of the resulting swim speed, we favored accuracy of the mean over accuracy of the variance). The model was run in hydrostatic mode, as tests in nonhydrostatic mode showed no improvement in calibration and substantially longer run times compared to hydrostatic simulations. Sensitivity tests also showed that a prescribed parabolic eddy viscosity yielded better calibration than the typically used Mellor-Yamada 2.5 (MY2.5; [42]) turbulence closure.

For analysis purposes we defined the mean river velocity, *u*_*river*_*(t)*, as the average downstream velocity averaged over the portion of the study area above the junction (consistent with the analysis region described below in the hydrodynamic calibration results). Discharge data, *Q*_*MSD*_, was taken from the Mossdale gauge and stage data from the Head of Old River gauge. Stage data was then combined with the DEM and clipped to the analysis region to estimate the time-varying water volume in the region, *V*_*a*_*(t)*. The characteristic length for flow through the region was *L*_*a*_ = 234 m, estimated from GIS as an average across flow paths leaving either branch of the junction. The mean river velocity was then calculated as

$$u_{river}=Q_{MSD}L_{a}/V.$$ (3)

While mean velocity data is available directly from the Mossdale gauge, the reach velocity is a function of cross-sectional area and this method provided a river velocity consistent with the rest of the analysis.

### Swimming velocity estimation

Fish velocity over ground, ***u***_*og*_, was calculated for each interval between successive relocations of each acoustic tag (termed a *segment*) by dividing the vector displacement by the intervening time interval. For each segment, the hydrodynamic velocity, ***u***_*h*_, was extracted from the model output at the spatial and temporal midpoint of the segment. Swimming velocity ***u***_*s*_ was defined as the vector difference between the velocity over ground and hydrodynamic velocity:

$$u_{s}\equiv u_{og}-u_{h}.$$ (4)

The multilateration procedure did not include a vertical coordinate, leaving some ambiguity around the appropriate three-dimensional hydrodynamic velocity to use in determining swim velocity. To understand how vertical distribution affects estimated swim velocities, we tested three potential assumptions for the vertical distribution of smolts: 1) uniform over the top 1 m of the water column; 2) uniform over the top 2 m of the water column; and 3) evenly distributed from the surface to the bed. For the calculation of swimming velocity, we extracted hydrodynamic velocities averaged over these same vertical ranges. The first two choices reflect a hypothesis that smolts are primarily surface-oriented [32, 43], while the depth-averaged velocity reflects an assumption that smolts are evenly distributed in the water column. Tags with mean swimming speed above a threshold of 0.5 m s^-1^ (one tag) were eliminated as likely predators, and not included in later analyses.

Swim velocity vectors were rotated into the coordinate frame of local flow to identify a longitudinal (positive downstream, negative upstream) swimming component *u*_*s*_ and a lateral (positive river right, negative river left) swimming component *v*_*s*_. Orientation of the local flow was defined by depth-averaged hydrodynamic velocity. Most analyses of the lateral swimming component disregarded sign of the lateral swimming velocity and instead utilized the lateral swimming speed, |*v*_*s*_|.

The distributions across all fish of longitudinal swimming (parallel to the flow) and lateral swimming (perpendicular to the flow) were summarized by kernel density estimates, one for each of the three vertical averaging ranges. Given the potential for swimming to bias number of segments available for an individual (e.g. a downstream-swimming individual would spend less time in the study area than an upstream-swimming individual), velocity estimates were weighted by the inverse of the number of segments for the respective individual. For each depth-averaging method the 5–95% confidence intervals for median longitudinal swimming was calculated with bootstrapping, drawing from the same weighted samples as used for kernel density estimates.

### Swimming velocity analysis

Swimming velocity was modeled as a smooth function of environmental variables using a generalized additive model (GAM), as implemented by the bam method of the mgcv library [44]. Temporal autocorrelation within tracks was modeled with a first-order autoregressive (AR(1)) structure. Tested correlates were time of day, turbidity, mean river velocity, water depth, vorticity (ω_xy_), and hydrodynamic speed (‖***u***_*h*_‖). Vorticity in the *x-y* plane (effectively the lateral shear) is defined as

$$\omega_{xy}\equiv \frac{\partial v}{\partial x}-\frac{\partial u}{\partial y}.$$ (5)

where *u* is the hydrodynamic velocity in the *x* direction and *v* the hydrodynamic velocity in the *y* direction. Vorticity was extracted from the hydrodynamic model output using the same vertical averaging range as velocity. The gradients in (5) were calculated by central difference over a linearly interpolated velocity field. Lateral swimming speed was included as a correlate for longitudinal swimming velocity, and longitudinal swimming velocity was included for lateral swimming speed. Samples were weighted by the inverse of the number of segments for the respective individual as described above. Results of the three vertical averaging ranges were treated as replicate measurements of each track and represented uncertainty in the data related to vertical position (an additional weighting factor of 1/3 was included to avoid inflating the degrees of freedom in the data). We did not include a random effect for individuals in the model, as many correlates had minimal variance within each track, such that an individual random effect would have led to a lack of identifiability. Time of day was smoothed with a cyclic cubic spline with a 24-hour cycle. All other terms were smoothed with cubic regression splines and included a shrinkage term to aid model selection. Autocorrelation and uncertainty in swim velocity estimates and environmental parameters reduced effective degrees of freedom (EDF) in the input data. We accounted for this by adjusting the gamma parameter to mgcv, selecting the minimal gamma for which no single smooth had more than four effective degrees of freedom. We selected this threshold to balance sufficient curvature to capture simple nonlinear effects while also avoiding unrealistically complex smooths. Terms were deemed not significant if *p*>0.05 or effective degrees of freedom decreased to <0.5.

While the analysis described above was focused on isolating swimming behaviors, it was also of interest whether the net effect of these behaviors led to downstream movement faster or slower than the average water velocity. This is important, for example, in estimation of transit times through a system or in one-dimensional transport models. Longitudinal swimming has a clear and direct contribution to downstream movement, but positioning within the cross-section towards faster or slower regions of the flow also alters how quickly an individual moves downstream. We summarized the net effect of longitudinal swimming and cross-sectional positioning by comparing each smolt’s downstream velocity over ground (averaged over the analysis region) to the simultaneous mean river velocity in the reach. The mean difference between the respective velocities was tested by a two-sided, paired t-test. Unlike the direct analysis of swimming velocities, this comparison does not involve any assumptions of vertical position, is entirely independent of the quality of the hydrodynamic calibration, and is affected only slightly by telemetry errors.

## Results

### Telemetry processing

**Fig 4:**
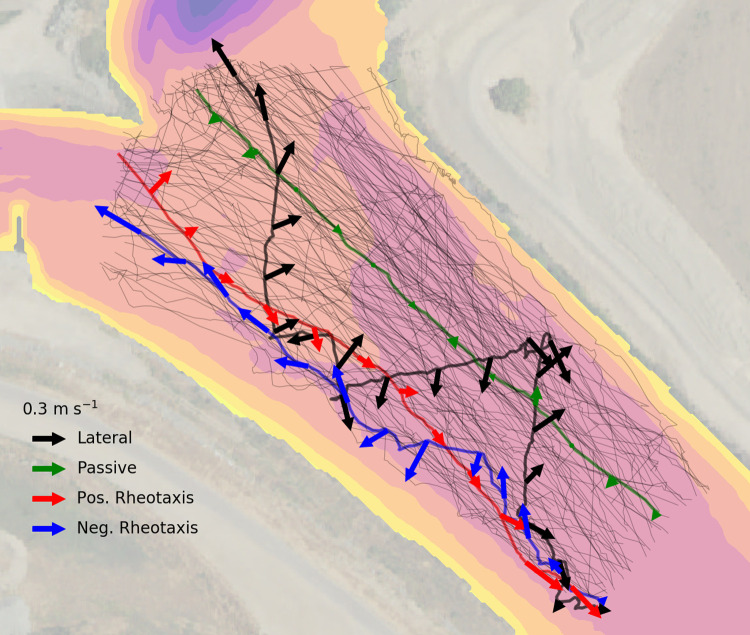
Fish tracks and behavior examples. All valid tracks within the analysis region (light lines), with four individuals highlighted as examples of specific behaviors. All tracks represent downstream movement from the lower right to the upper left. Background contours show depth.

The acoustic array produced a total of 2.7 million detections during the period 2018-03-13 to 2018-04-15 (after removal of likely multipath receptions). The majority of those detections were from beacon-to-beacon synchronization pings, with 147,991 receptions from fish tags. Of the 650 tagged fish, 348 were detected at least once in the array, 132 (38%) from the upper release and 216 (62%) from the lower release. After estimating fixed station locations and applying the external clock offset corrections, the remaining position estimates had a median uncertainty of 1.42 m (YAPS-reported standard deviation of position), with an interquartile range of 0.43–2.57 m. One tag that satisfied earlier screening criteria was deemed to be a predator based on mean swimming speed = 0.86 m s^-1^, nearly double the next fastest value. After screening criteria, 121 tags yielded valid tracks and were used in remaining analyses (Fig 4).

### Hydrodynamic model calibration

**Fig 5:**
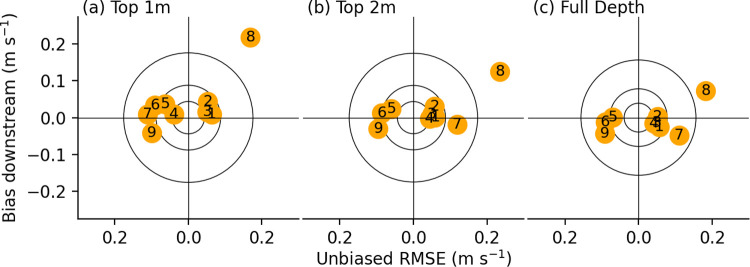
Hydrodynamic model calibration target diagrams. Target diagrams depicting model skill per transect for the three vertical averaging ranges. Transects are numbered 1 (most upstream) to 7 (junction), 8 (Old River), and 9 (San Joaquin River, downstream of junction). Points nearer the center have less error, with a vertical offset from the center denoting bias (i.e. systematic error), and a horizontal offset from center denoting unbiased root mean square error (uRMSE, i.e. random error). Predicted variance dictates whether points fall left of center (underpredicted variance) or right of center (overpredicted variance). Circles show the scale of 0.25, 0.5, and 1.0 standard deviations (calculated across all transects). Transects 8 and 9 are shown but correspond to portions of the domain that were not used in the analysis.

Hydrodynamic velocity calibration results are summarized in Table 1 and in target diagrams in Fig 5. While target diagrams [45] are often normalized by the standard deviation of the observations [46], here we retain the dimensional values of (1) and (2) to aid in comparing the scales of model uncertainty and inferred swimming velocities (Fig 5). Multiple calibration metrics are presented for each transect, and averaged over three portions of the vertical coordinate (top 1 m, top 2 m, and full water column). Taking the average across the upper six transects and across all three vertical intervals, the mean uRMSE was 0.06 m s^-1^ (0.06 m s^-1^ for longitudinal, 0.02 m s^-1^ for lateral), and the mean biases were 0.009 m s^-1^ and 0.001 m s^-1^ for, respectively, longitudinal and lateral velocities.

**Table 1:** Metrics for hydrodynamic calibration.

Transect	Slice	uRMSE (m s^-1^)	uRMSE (u) (m s^-1^)	uRMSE (v) (m s^-1^)	BIAS (u) (m s^-1^)	BIAS (v) (m s^-1^)
**1**	Full Depth	0.056	0.054	0.016	-0.026	-0.005
	Top 1m	0.063	0.062	0.009	0.009	0.011
	Top 2m	0.060	0.056	0.019	0.000	-0.003
**2**	Full Depth	0.051	0.048	0.017	0.002	-0.002
	Top 1m	0.053	0.052	0.009	0.043	0.006
	Top 2m	0.057	0.055	0.017	0.031	-0.002
**3**	Full Depth	0.048	0.045	0.017	-0.013	0.007
	Top 1m	0.050	0.050	0.006	0.015	-0.004
	Top 2m	0.050	0.046	0.020	0.009	0.003
**4**	Full Depth	0.042	0.036	0.021	-0.015	0.004
	Top 1m	0.040	0.040	0.004	0.009	-0.004
	Top 2m	0.044	0.039	0.021	-0.002	0.004
**5**	Full Depth	0.070	0.069	0.015	0.001	0.006
	Top 1m	0.063	0.062	0.009	0.038	-0.006
	Top 2m	0.060	0.058	0.014	0.024	0.003
**6**	Full Depth	0.091	0.072	0.055	-0.012	0.002
	Top 1m	0.091	0.091	0.012	0.034	-0.004
	Top 2m	0.087	0.067	0.055	0.012	0.002
**7**	Full Depth	0.111	0.085	0.071	-0.048	-0.023
	Top 1m	0.111	0.103	0.042	0.010	-0.001
	Top 2m	0.118	0.095	0.071	-0.019	-0.025
**8**	Full Depth	0.183	0.169	0.070	0.073	0.054
	Top 1m	0.169	0.161	0.051	0.218	0.029
	Top 2m	0.234	0.214	0.096	0.125	0.080
**9**	Full Depth	0.092	0.085	0.035	-0.042	0.000
	Top 1m	0.100	0.085	0.053	-0.041	0.054
	Top 2m	0.096	0.088	0.040	-0.030	0.030

Overall the model slightly overpredicts near surface velocities, and diverges from the observations below the junction. Due to the complex flows around a scour hole on the San Joaquin below the junction and the uncertain configuration of the partial barrier on the Old River branch, the model performs poorly below the junction. Most hydrodynamic models at this scale make a hydrostatic assumption whereby vertical momentum of flow is neglected. While boils were observed at the surface near the scour hole, which suggests a breakdown of this hydrostatic assumption [47], nonhydrostatic simulations did not show significant improvement in calibration. This may be due to insufficient resolution to capture these motions or the evolving geometry of the scour hole and adjacent sand bar. Given the model performance below the junction, the analysis in the remainder of the paper focuses on currents and fish behavior leading up to the junction.

**Fig 6:**
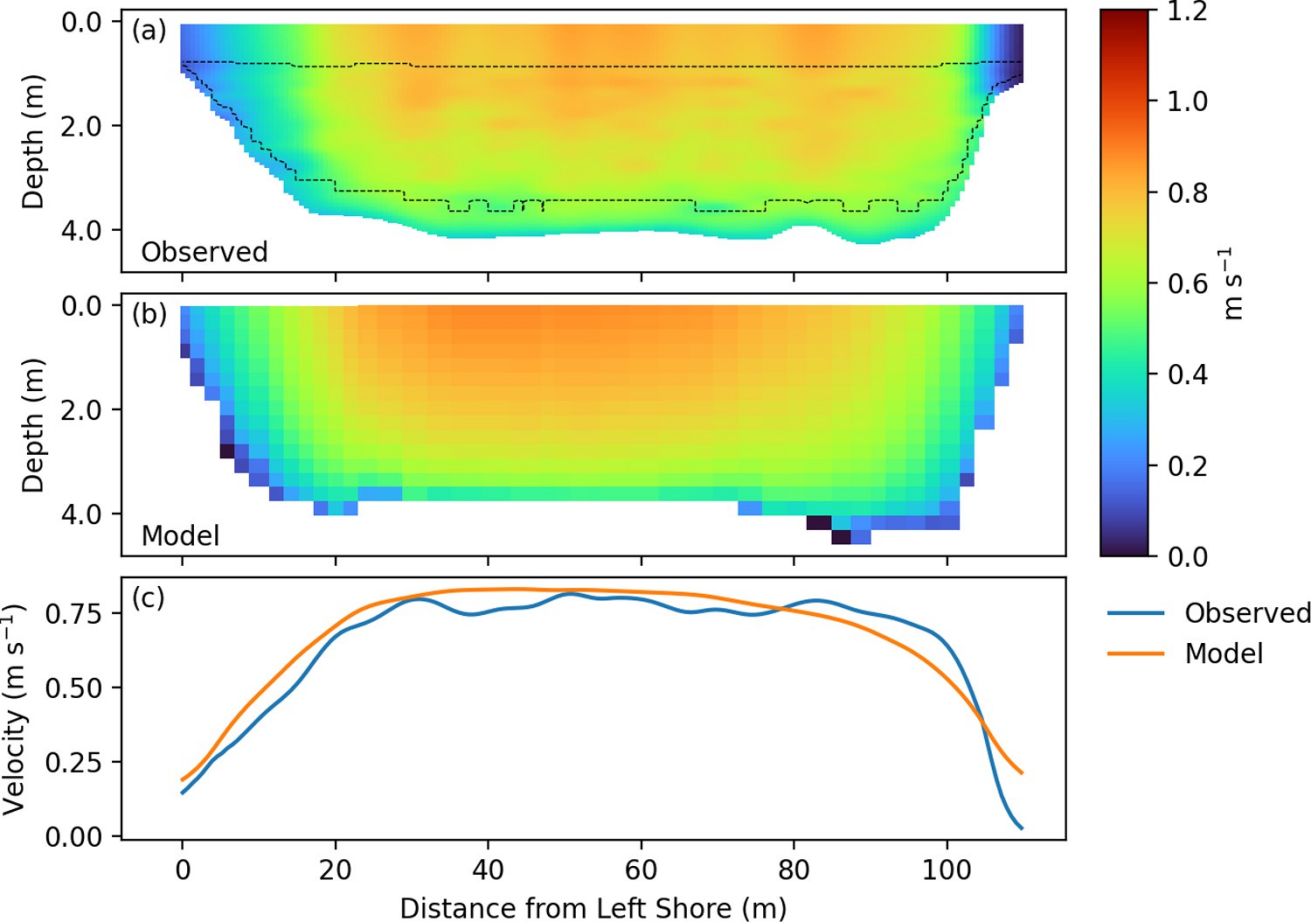
Model–data comparison for transect 5. Velocity comparison between ADCP observations (a) and model predictions (b) at transect 5. Velocity integrated over the top 2 m of the water column is plotted in (c). Dashed line in top panel denotes the extent of the original ADCP data before extrapolation. Field data has been smoothed with Hann filters of window size 9 in the vertical and 15 in the horizontal.

Fig 3 shows a plan-view comparison between the model and observations at each of the transects, demonstrating accurate resolution of the flow direction and magnitude above the junction. The largest errors are in the highly-sheared portions of the flow near shoreline, and in complex flows below the junction. Fig 6 shows a sample cross-section at transect 5, where the model captures both the lateral and vertical velocity distributions reasonably well, with the minor exception of the shear near the right bank (Fig 6c). Secondary flow was weak in the transects above the junction. During low flow conditions before 2018-03-24, flow on the San Joaquin River below the junction reversed direction with most flood tides (Fig 2). Flow above the junction and on Old River was ebb-directed during the entire field campaign, and during high flow periods all flow was ebb-directed (as in Fig 3).

### Swimming velocity estimation

**Fig 7:**
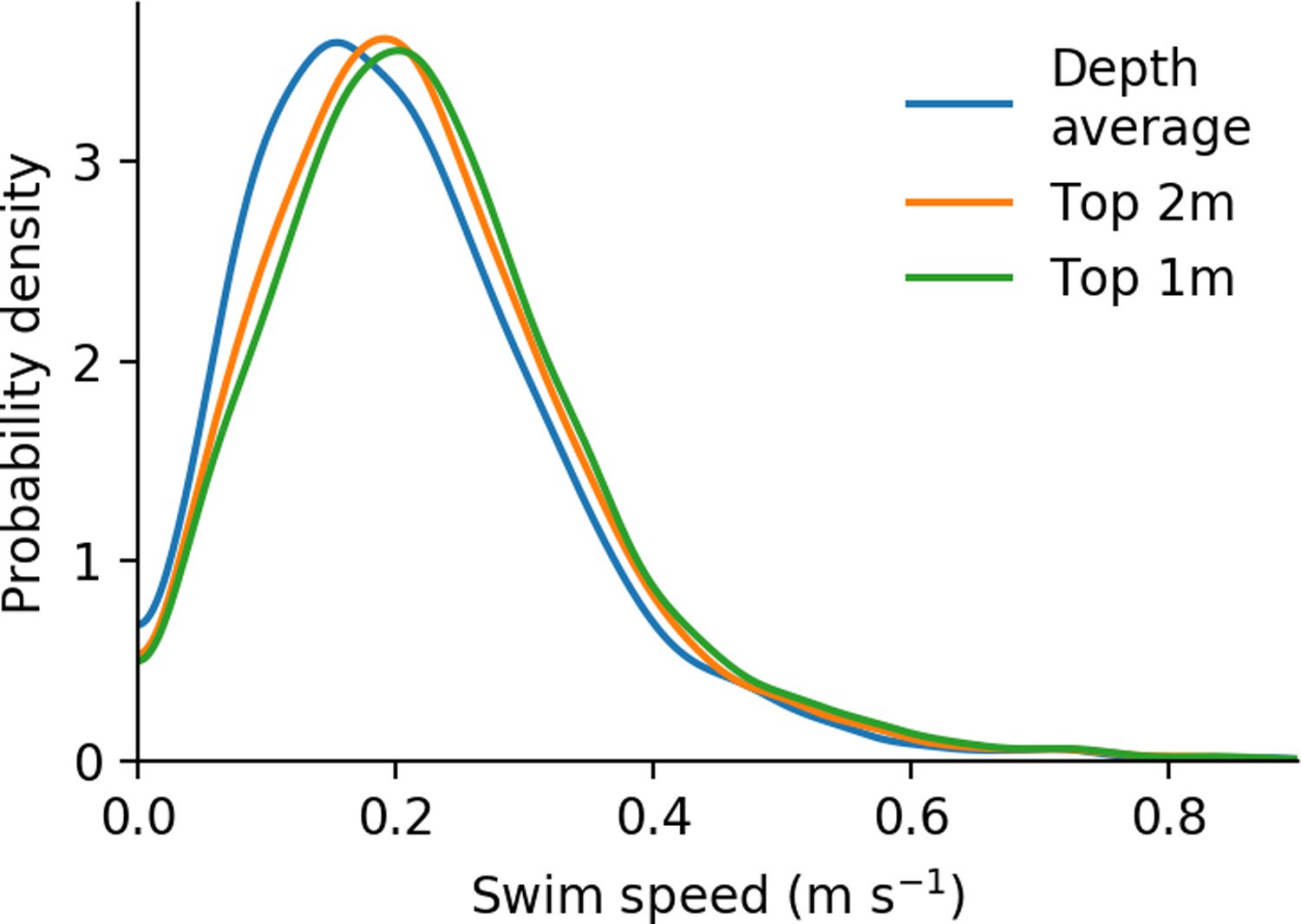
Distributions of swimming speed. Kernel density estimate of swimming speeds over all segments in the analysis region, relative to three assumptions of vertical distribution.

Distributions of resulting swim speeds (i.e. the magnitude of the difference between velocity over ground and hydrodynamic velocity), are plotted in Fig 7. The three distributions shown correspond to three choices of vertical averaging when extracting hydrodynamic data, which in turn reflect the three assumptions of how smolts are vertically distributed in the water column. Modes of distributions were 0.15–0.20 m s^-1^, corresponding to 2.0–2.7 BL s^-1^, respectively. Swimming speed calculated relative to depth-averaged hydrodynamic velocity was the slowest, and swimming speed relative to the top 1 m the fastest. Average swimming velocities were slightly biased towards positive rheotaxis, such that for a given observed velocity over ground, a faster hydrodynamic velocity (i.e. top 1 m water velocity) implies a faster swimming velocity. The 0.05 m s^-1^ range of the modes is an approximate measure of the uncertainty in swim speed due to unknown vertical position of tagged fish.

**Fig 8:**
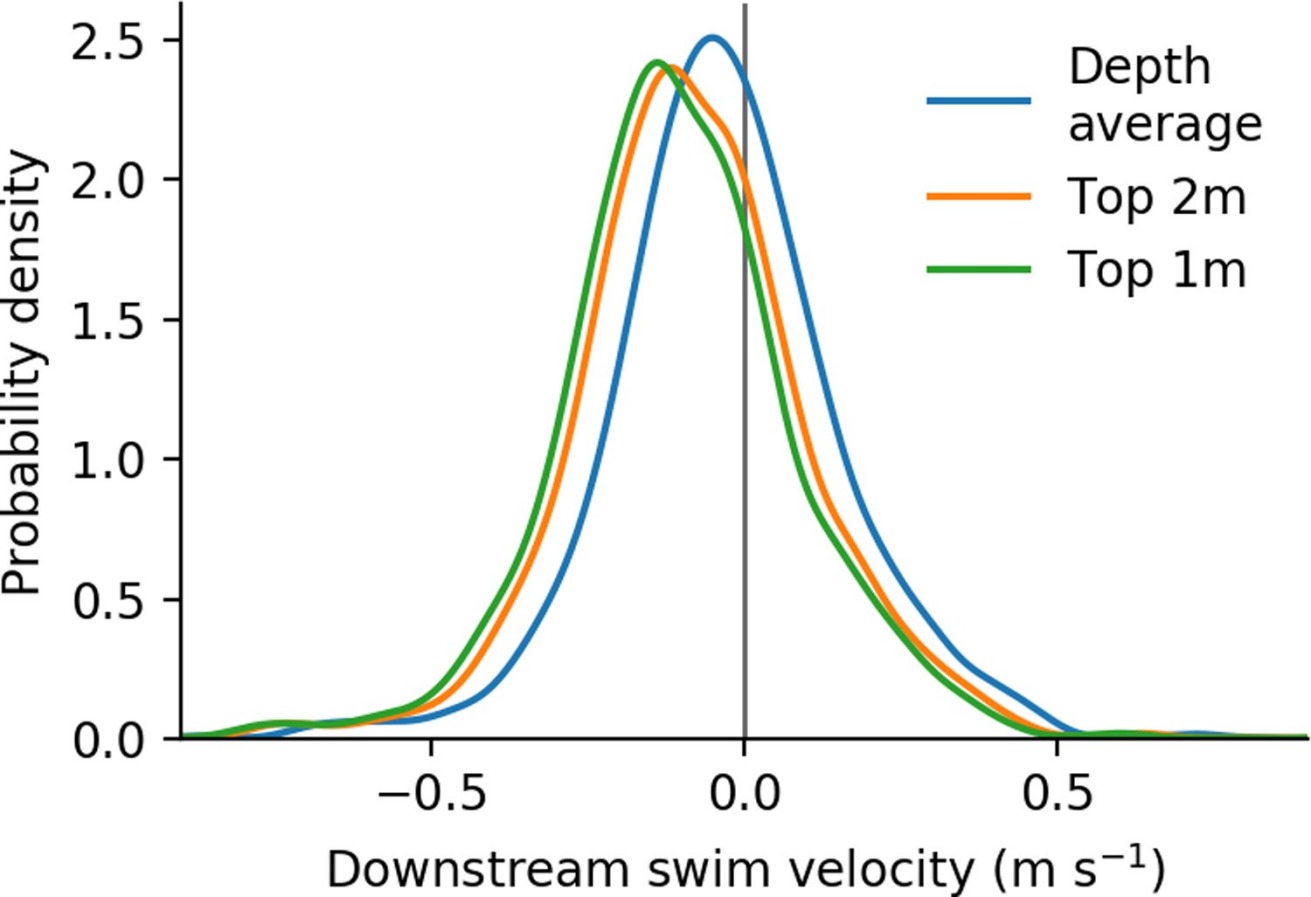
Distributions of longitudinal swimming velocity. Kernel density estimate of longitudinal swimming velocity, normalized to equally weight each individual. Negative values indicate positive rheotaxis (i.e. swimming opposite the flow).

The distribution of longitudinal swimming velocity is shown in Fig 8. Summary statistics of the distributions are shown in Table 2. Regardless of vertical average the median downstream swimming velocity was negative (i.e. positive rheotaxis), including the full range of the 5–95% confidence interval for the median. The distribution of lateral swimming velocity was only weakly affected by choice of vertical averaging, with the median value approximately zero for all choices of vertical averaging. Median lateral swimming speed was 0.090 m s^-1^ for all vertical averaging ranges (5–95% confidence interval 0.086–0.093 m s^-1^). Individuals exhibited distinct and often persistent behavior including positive rheotaxis, negative rheotaxis, passive transport, and lateral swimming. Examples of tracks exhibiting these four primary behaviors are highlighted in Fig 4 among the full set of observed tracks. This figure also shows the footprint of the analysis area, covering an area of 25,200 m^2^ along the 240 m long reach.

**Table 2:** Summary statistics of longitudinal swimming.

Vertical average	Median downstream swim velocity	Occurrence of positive rheotaxis
	*m s*^*-1*^ *[5–95% c*.*i*.*]*	
Depth average	-0.036 [-0.042, -0.031]	59%
Top 2m	-0.090 [-0.096, -0.084]	70%
Top 1m	-0.113 [-0.118, -0.108]	74%

### Swimming velocity analysis

**Fig 9:**
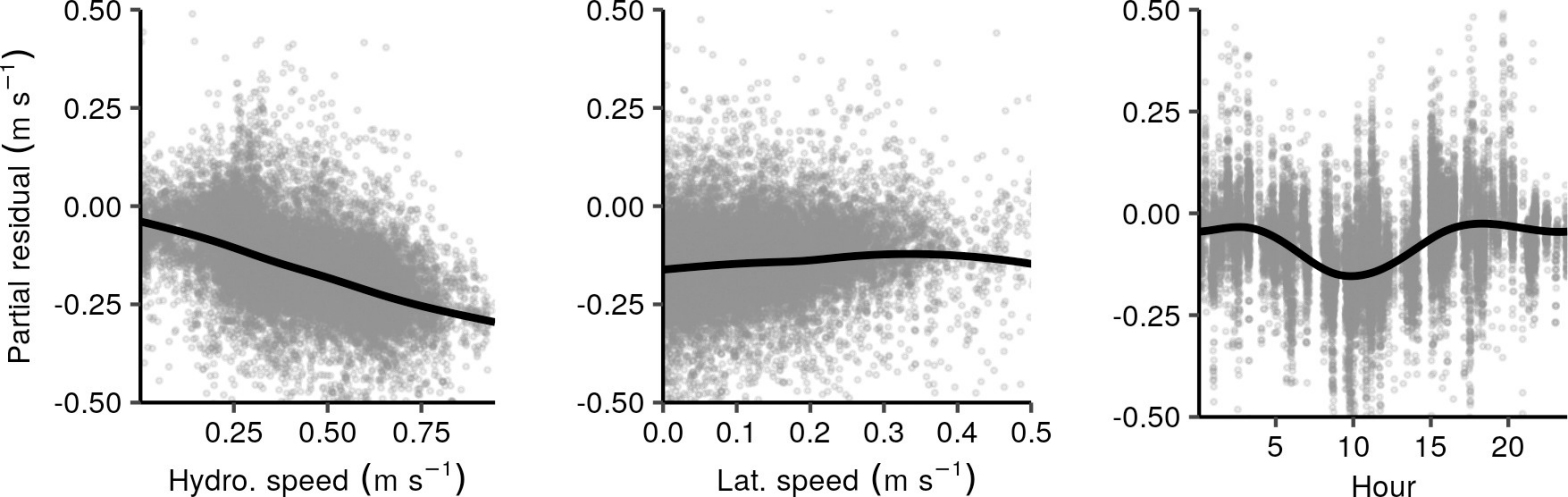
Summary of GAM for longitudinal swimming velocity. Significant smooths (black line) and partial residuals (gray circles) for GAM of longitudinal swimming velocity.

The GAM fit for longitudinal swimming is summarized in Table 3. Of the tested correlates, hydrodynamic speed, lateral swim speed, and time of day were found to be significant. The respective smooth functions and residuals are shown in Fig 9. Positive rheotaxis was inversely related to lateral swimming (aside from a reversal of this trend in the upper tail of the lateral swimming distribution). In other words, longitudinal swimming trended from upstream swimming towards zero as lateral swim speed increased, possibly indicating that fish generally adopt a swim speed independent of choice of direction. Upstream swimming was also correlated with hydrodynamic speed with individuals tending to swim opposite, but not faster than, the local current. The smooth for time of day indicated a diel pattern spanning 0.13 m s^-1^ with maximum positive rheotaxis near 10:00 local time. Time of day was the only significant term with minimal within-track variation and the only smooth lacking a shrinkage term. As such, its statistical power may be overstated by the reported EDF and p-value.

**Table 3:** Summary of GAM for longitudinal swimming velocity.

Independent variables	EDF	P
**Local water speed**	2.5	< 10^−15^
**Lateral swim speed**	3.8	< 10^−15^
Vorticity	5.5×10^−6^	0.41
Depth	9.1×10^−6^	0.061
**Time of day**	3.9	< 10^−15^
Turbidity	5.2×10^−5^	8.7×10^−4^
River velocity	1.4×10^−5^	0.035

Gamma = 11. Significant terms are in bold.

**Fig 10:**
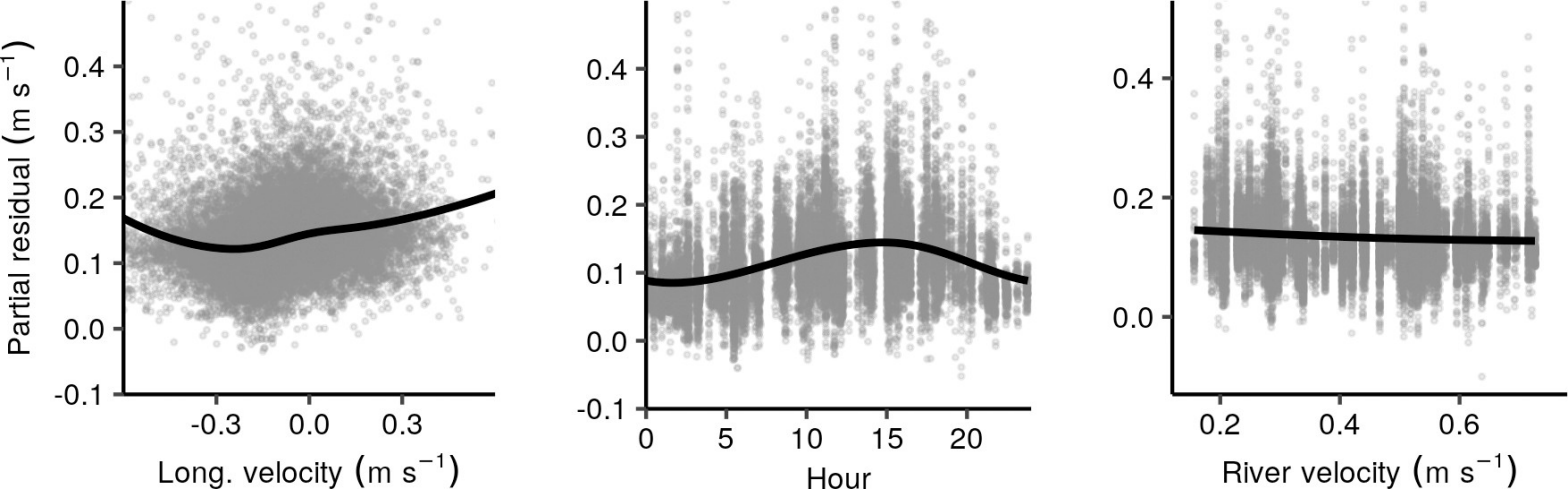
Summary of GAM for lateral swimming speed. Significant smooths (black line), and partial residuals (gray circles) for GAM of lateral swimming speed.

The GAM for lateral swimming speed is summarized in Table 4. Longitudinal swimming velocity, time of day, and river velocity had significant relationships with lateral swimming speed. The respective smooth function and residuals for each significant term are shown in Fig 10. Dependence of lateral swimming on longitudinal swimming was slightly more complex than the opposite dependence described above. With the exception of strongly positive rheotaxis (u<-0.25 m s^-1^), lateral swimming was faster for increasingly downstream longitudinal swimming. Lateral swimming was strongly related to time of day, with a clear diel pattern of faster lateral movement during daylight hours. Peak lateral swimming occurred near 14:00 local time.

**Table 4:** Summary of GAM for lateral swimming speed.

Independent variable	EDF	p
Local water speed	6.2×10^−6^	9.8×10^−4^
**Longitudinal swim velocity**	4.0	< 10^−15^
Vorticity	2.2×10^−6^	0.20
Depth	3.4×10^−6^	5.1×10^−3^
**Time of day**	2.4	< 10^−15^
Turbidity	2.3×10^−6^	0.13
**River velocity**	0.62	< 10^−15^

Gamma = 33. Significant parameters are in bold.

Downstream velocity over ground was compared to mean river velocity (Eq 3) to understand the net effect of behavior on downstream movement. Downstream velocity over ground had a mean of 0.42 m s^-1^ and median 0.42 m s^-1^, and river velocity had a mean of 0.47 m s^-1^ and median 0.51 m s^-1^. The mean difference between velocity over ground and mean river velocity was distinct from zero (N = 121, p = 0.00024) with a value of 0.05 m s^-1^ (90% confidence interval 0.02 to 0.07 m s^-1^). This demonstrates that behavior effectively slowed the downstream movement of smolts.

## Discussion

Spring-run Chinook Salmon have shown increased population growth in San Joaquin River in recent years (attributed to restoration programs). For example, numbers of spawning adult and numbers of observed redds have increased dramatically since 2017 [48]. Over the 2017–2018 winter and spring approximately 200,000 juvenile spring-run Chinook Salmon were released into the San Joaquin River [49]. However, survival rates through the Delta, often less than 5%, diminish the possibility of a self-sustaining population [50]. Designing and evaluating potential management actions calls for a mechanistic understanding of fish movement, informed by *in situ* observations of fish movements such as those presented in the present analysis. In particular, agent-based models and individual-based models should use this information in fish swimming representations.

The range of estimated swimming speeds was centered around 2–3 BL s^-1^ (0.15–0.20 m s^-1^). For all three choices of vertical averaging range the swimming speeds are similar to, but slower than, previously observed swim speeds for juvenile Chinook Salmon, such as the approximate 4 BL s^-1^ (0.28 m s^-1^) annular flume measurement from [9] (as interpolated for 70 mm fish, 0.40 m s^-1^ flows, and averaged over day–night conditions). The long tail of greater velocities is also comparable to the result of [21] who measured maximum sustained swim speeds of approximately 7 BL s^-1^ (approximately 0.60 m s^-1^). The difference in the presently observed swimming speeds and previously quantified maximum swimming speeds is consistent with emigrating fish choosing a slower, energy-conserving, swimming speed during most of emigration. A broad range of swimming behaviors (positive and negative rheotaxis, lateral swimming, and passive transport) were observed, and in many cases one fish exhibited multiple types of behavior. Though the tagged fish were emigrating towards the ocean, the most prominent behavior was positive rheotaxis.

### Bias and uncertainty

Quantitative interpretation of swimming velocity of tagged fish and the relationship of velocity to environmental factors requires careful consideration of bias and uncertainty. Swimming velocity was estimated as the vector difference of fish velocity over ground and hydrodynamic velocity. Both of these quantities are larger than typical swim velocities and accuracy in both are required in order for swim velocities to be meaningful. Bias and uncertainty in swimming velocity estimates arise from telemetry uncertainty, hydrodynamic model and observation error, and the vertical position of fish.

The YAPS package [37] allowed substantial improvements in accuracy relative to simpler approaches by estimating all positions simultaneously, including an estimate of the position uncertainty. A commercial, proprietary multilateration method (similarly using time difference of arrival but analyzing each ping independently) put 9.6% of tag locations on dry land, compared to 0.5% for YAPS (for positions calculated with three or more receivers). Removing tag locations based on the reported uncertainty was the only filtering required. The accuracy was achieved primarily due to the sophistication of the YAPS algorithm, aided by an alternative hydrophone clock synchronization approach that avoided discontinuities in clock offsets during noisy, high-flow periods of the experiment. While some fidelity in calculated positions was lost as a result of separating the receiver location estimation and the clock drift estimation, this procedure reduced overall error by eliminating discontinuous clock offsets.

The power of YAPS notwithstanding, uncertainty in positions from telemetry arises from many sources including residual clock offsets, vertical hydrophone offsets, speed of sound errors, undetected multipath propagation, and hydrophone array geometry [37, 51]. Field data would ideally include tag drifts with high-precision GPS fixes such that telemetry errors could be directly quantified (at least for the flow conditions during the tag drift). In the present study tag drifts were attempted in a second, later deployment, but high flows rendered the telemetry results unusable. Error analysis was thus limited to qualitative and semi-quantitative approaches.

Bias in positions from telemetry was assumed to be negligible, and would affect swimming velocities only to the degree that hydrodynamic data would be extracted from a point offset from the true position. In contrast, random position uncertainty may influence estimated speed over ground. YAPS reported a median position uncertainty of 1.4 m. If position errors were uncorrelated then the error in velocity over ground (over a 5 s ping interval) would be of the same magnitude as typical swimming speed estimates. However, we expect most of the sources of error in telemetry positions are substantially autocorrelated at the time scale of successive pings, with the notable exception of multipath errors. Individual velocity and speed estimates depend only on relative change in position such that errors cancel out as the error autocorrelation approaches one. Mean velocity is also immune to uncorrelated position error when the mean is over a sufficiently long period. However, uncorrelated errors still affect individual velocity and speed estimates and inflate the respective variances.

Unlike vector quantities, estimated speeds (e.g. lateral swimming speed, or speed over ground) gain a bias towards greater values in the presence of uncorrelated errors in telemetry, in addition to the inflated variance mentioned above. Of the reported swimming metrics, swim speeds (Fig 7) were most sensitive to noise in telemetry results. We did not have direct measurements of the error autocorrelation, which would allow a calculation of what portion of the YAPS-reported errors were uncorrelated. Instead, we repeated swim speed calculations as in Fig 7 but using multiple strides over the original samples as an approximate measure of the role of uncorrelated errors. When considering swim speed estimates calculated at the original PRI of 5 s, the median swim speed was 0.216 m s^-1^, compared to 0.190 m s^-1^ when calculated over an interval of 40 s. The difference of 0.026 m s^-1^ is an estimate of the scale of the combined effects of uncorrelated telemetry error and sinuosity of the true paths of the tags. The respective standard deviations were 0.134 m s^-1^ and 0.115 m s^-1^, suggesting that the distribution in Fig 7 may also be slightly wider than the true distribution.

Swimming speed and velocity estimates were also susceptible to bias and uncertainty in hydrodynamic model predictions. The hydrodynamic calibration process included estimates of bias and uncorrelated errors (in the form of uRMSE), relative to ADCP measurements. Bias was typically less than 0.02 m s^-1^, with a mean of less than 0.01 m s^-1^ for both longitudinal and lateral directions. uRMSE was 0.06 m s^-1^ and 0.02 m s^-1^ for longitudinal and lateral velocities, respectively. Some additional uncertainty is due to the calibration taking place during flows that were greater than the flows when most tags passed through the array. While this uncertainty is difficult to quantify, we expect bias would not change substantially and uRMSE would be smaller during low flow periods. The fine resolution used in the model in this study was also critical in resolving nearshore velocity gradients and allowing reliable and consistent swim speed estimates even in shallow nearshore waters. Even in the worst case combination of telemetry errors (0.03 m s^-1^) and hydrodynamic bias (0.02 m s^-1^), the potential net bias in estimated swimming speed is still small relative to the median estimated swimming speed.

Vertical position and movement of fish also affect bias and uncertainty in estimated swimming velocities. The use of a well-calibrated three-dimensional hydrodynamic model allowed us to estimate uncertainty and bias in swimming velocities associated with unknown but stationary vertical distributions of fish. For example, we found a 0.077 m s^-1^ difference between estimated median swim speeds when assuming fish were in the top meter versus evenly distributed vertically. Using a depth-averaged hydrodynamic model would have underpredicted positive rheotaxis if the fish were in fact surface-oriented. At short time scales (seconds to minutes) vertical movement also contributes directly to true swimming speed, and the omission of this term in our analysis could lead to a bias toward slower swim speeds. Few observational data are available on the vertical movement of Chinook Salmon smolts at these time scales. However, observations of Atlantic salmon post-smolts [52] suggest that vertical swimming speed was generally less than 0.017 m s^-1^. To the extent that these data are relevant for Chinook smolts in freshwater, we conclude that the contribution of vertical movement to swimming speed is small relative to horizontal movement. Systematic vertical movement over longer time scales, however, can lead to errors in estimated longitudinal swimming velocities due to the vertical variation of hydrodynamic velocity. If, for example, smolts occupied the top 1 m of the water column during the night and were distributed across the full depth during the day, then an assumption of a constant vertical distribution would lead to an *apparent* pattern of faster upstream swimming during the day. The scale of this effect is approximately the range of median longitudinal swimming velocities reported in Table 2: 0.077 m s^-1^. With this in mind, the diel pattern in Fig 9 may in fact be due to vertical diel migration rather than changes in horizontal swimming behavior.

While the current study achieved substantial accuracy in measuring swimming speeds, the lack of vertical distribution data was a clear limitation. This could be remedied in future studies by prioritizing vertical resolution over horizontal coverage when designing the hydrophone array. A denser array with a smaller horizontal footprint may also allow for some or all of the array to be cabled together such that clock synchronization could be handled over a local, hard-wired connection, further improving the system’s accuracy.

### Interpretation and applications

Swimming behavior leads to a difference in how quickly smolts move downstream relative to the average velocity of the water, and the results show that smolt movement was slower than the water velocity. In the absence of swimming and regulation of vertical position, individuals would be passively transported with the flow, evenly distributed across the cross-section of the channel. Net downstream movement of smolts may depart from the mean river velocity (Eq 3) from either the direct effect of rheotaxis (i.e. positive rheotaxis retarding downstream movement) or from swimming placing the fish in faster or slower than average parts of the flow (e.g. greater velocity mid-channel and near-surface). Net downstream movement of fish was slower than the mean river velocity by 0.09 m s^-1^, consistent with observations of positive rheotaxis. While the difference is consistent with our estimate of rheotaxis (median of 0.09 m s^-1^ considering the top 2 m of the water column), we note that in the present analysis the effects of rheotaxis and positioning in the cross-section remain conflated. Nevertheless, this comparison provides an additional check on the inferred swimming velocities, independent of the hydrodynamic model, and also provides a measure of how swimming affects migration in the aggregate.

Lateral swimming speed varied with time of day, with faster lateral swimming during daylight hours. This may be a searching behavior, where fish are attempting to find cover in shallow, near-shore habitat during times when visibility and predation risk are greatest. The highly engineered channels of the Sacramento-San Joaquin Delta generally provide little cover [53–55], such that a search for cover is often unsuccessful. If such searches were typically successful we would expect to see decreased downstream velocity during daylight hours while fish sheltered in low-velocity perimeters. However, such a signal was not present in the data. Further building on this hypothesis, one can imagine selectively restoring suitable habitat on one bank or the other in order to shape the lateral distribution of fish along a reach, and ultimately alter route selection. Along similar lines, the magnitude of lateral swimming has implications for how lateral distributions of fish change as they migrate downstream. Median lateral swimming speed (0.09 m s^-1^) and downstream velocity over ground (0.42 m s^-1^) can be used to approximate the rate at which lateral distributions are homogenized by swimming. These results can be used to evaluate whether, for example, the effects of a river bend (such as in [32]) will persist long enough to affect entrainment at a downstream junction. Improved data on lateral swimming may also aid the generalization of results from studies such as [24], by quantifying aspects of fish behavior that affect the persistence of cross-sectional distributions along a reach. Lastly, habitat restorations and nonphysical barriers may affect lateral distributions [56], and alterations to route selection may depend on persistence of changes in lateral distribution.

## Conclusion

The swimming behavior of emigrating Chinook Salmon smolts is an important factor in route selection and transit time. Overall survival of smolts transiting the branching channel network of the Sacramento-San Joaquin Delta varies by route, and various engineering approaches such as physical and non-physical barriers have attempted to shape route selection. Understanding swimming behavior is essential for effective modeling and management of this system, as well as robust interpretation and extrapolation of telemetry data. We combined two-dimensional telemetry of tagged hatchery fish with a high resolution hydrodynamic model to quantify the range of swimming behavior of emigrating salmon smolts at a tidal junction. Employing a recently developed telemetry solver greatly increased fidelity of tag positions. When combined with a carefully calibrated hydrodynamic model, the uncertainty in swimming velocity was small relative to the estimated velocities. Mean swim speeds were highly variable with a median near 0.20 m s^-1^. Positive rheotaxis was broadly observed, increased with water velocity, and was consistent with smolts moving through the area more slowly than the mean flow velocity. Diurnal variation in longitudinal swimming indicated greater positive rheotaxis or downward vertical migration during daylight hours. Lateral swimming was common and most prevalent during daylight hours, suggesting a searching behavior when predation risk was higher than at night.
